# Detection limits of *Legionella pneumophila* in environmental samples after co-culture with *Acanthamoeba polyphaga*

**DOI:** 10.1186/1471-2180-13-49

**Published:** 2013-02-26

**Authors:** Lisa Conza, Simona Casati, Valeria Gaia

**Affiliations:** 1 Swiss National Reference Centre for Legionella, Cantonal Institute of Microbiology, Via Mirasole 22a, 6500, Bellinzona, Switzerland

**Keywords:** *Legionella*, Culture, Co-culture, Compost, Air, Bioaerosol, Detection limit

## Abstract

**Background:**

The efficiency of recovery and the detection limit of *Legionella* after co-culture with *Acanthamoeba polyphaga* are not known and so far no investigations have been carried out to determine the efficiency of the recovery of *Legionella* spp. by co-culture and compare it with that of conventional culturing methods. This study aimed to assess the detection limits of co-culture compared to culture for *Legionella pneumophila* in compost and air samples. Compost and air samples were spiked with known concentrations of *L*. *pneumophila*. Direct culturing and co-culture with amoebae were used in parallel to isolate *L*. *pneumophila* and recovery standard curves for both methods were produced for each sample.

**Results:**

The co-culture proved to be more sensitive than the reference method, detecting 10^2^-10^3^ *L. pneumophila* cells in 1 g of spiked compost or 1 m^3^ of spiked air, as compared to 10^5-^10^6^ cells in 1 g of spiked compost and 1 m^3^ of spiked air.

**Conclusions:**

Co-culture with amoebae is a useful, sensitive and reliable technique to enrich *L. pneumophila* in environmental samples that contain only low amounts of bacterial cells.

## Background

The genus *Legionella* includes approximately 53 species [1], with *Legionella pneumophila* being the most common human pathogenic species and causing 90% of all outbreaks of Legionnaires’ disease (LD) in Europe [2]. *Legionella* species are ubiquitous microorganisms, occurring predominantly in aquatic environments, freshwaters and hot water systems [2], soils, potting soils [3], and composts [4]. Cooling towers, whirlpool spas and shower faucets could be the sources of contaminated bioaerosols, the inhalation of which is generally considered to cause LD outbreaks [2].

A variety of culture methods to detect *Legionella* species are used to analyze environmental samples [5]. Experience of the laboratory staff in *Legionella* identification mostly influences and limits the sensitivity of the method [2]. For clinical samples, for instance, the sensitivity and specificity of culture for respiratory secretions are approximately 42.8% and 100%, respectively [5,6]. The standard detection method (ISO/DIS 11731) for *Legionella* in environmental samples consists of inoculating samples on selective glycine–vancomycin–polymyxin B–cycloheximide (GVPC) agar or on non-selective buffered-charcoal-yeast-extract (BCYE) [5,7]. Limitations of the plating method are prolonged incubation periods [5,8]; bacterial losses due to sample centrifugation or filtration and decontamination steps [8]; presence of contaminating microorganisms that may interfere with *Legionella* growth, thus decreasing sensitivity; and presence of *Legionella* cells as viable but not cultivable (VBNC) organisms [9]. The sensitivity of the culture method for samples with low *Legionella* counts (e.g. bioaerosols and rain) may be enhanced with an efficient enrichment or concentration step; correspondingly, samples with a rich and diverse flora (e.g. soils and composts) should be decontaminated before culture to inhibit growth of concurrent microorganisms [5], because the use of selective media cannot completely inhibit the growth of moulds, bacteria and yeasts [5].

Free-living amoebae (FLA) have long been used to enhance isolation of amoeba-resistant bacteria [10] and already more than 20 years ago Rowbotham proposed to use amoebal enrichment (co-culture) to recover *Legionella* from natural habitats and clinical specimens [11].

Co-culture aims to enrich the bacteria present in the specimen by exposing them to viable host amoebae [12]. The relative numbers of amoebae used for enrichment is important because too few amoebae may be destroyed before infection [13] and too many may encyst before spread, because *L. pneumophila* is able to penetrate trophozoites but not cysts [13]. Using co-culture, *Legionella* bacteria could be easily detected even in samples with high contaminant loads [12]. Macrophages have also been employed for enrichment steps [11].

*L. pneumophila* serogroup 1 strains are known to grow inside *Acanthamoeba* (*A. castellanii* and *A. polyphaga*) and *Naegleria*[14]. Non-pneumophila strains, e.g. *L. anisa*[12], *L. drancourtii*[15], *L. micdadei*[16], have also been isolated by co-culture with *A. polyphaga*.

Because of its sensitivity, the co-culture has the potential of improving bacterial yields in surveys of environmental samples with low *Legionella* counts or containing contaminating microorganisms. Co-culture has been described as the method of choice for the isolation of *Legionella* species, but no investigations have so far been carried out to compare the recovery efficiency for *Legionella* by co-culture with that of conventional culturing methods. In addition, the efficiency of recovery and the detection limit of *Legionella* after co-culture with *A. polyphaga* are not known.

In the present work, we utilized *L. pneumophila* as a model organism to study the interactions with *A. polyphaga* which, together with *A. castellanii*, is one of two FLA frequently used in co-culture experiments. We used trophozoites of the *A. polyphaga* because this species can be easily infected with *L. pneumophila* and can be effortlessly grown *in vitro*[13,14].

This study aimed to determine the detection limits of co-culture with *A. polyphaga* compared to conventional culturing methods for *L. pneumophila* in compost and air samples.

## Methods

### Bacterial and amoebal strains

*L. pneumophila* Philadelphia 1 (Lp1) strain (ATCC 33152) was grown on BCYE (bioMérieux, Geneva, Switzerland) at 36°C for 48 h, re-suspended and adjusted to 1.5 × 10^8^ CFU/ml in 2.5 ml of API® suspension medium (bioMérieux) with an ATB 1550 densitometer (bioMérieux) to prepare the dilutions to be used for spiking. One millilitre of serial dilutions of Lp1 suspension were prepared to obtain a range of 1 × 10 to 1 × 10^8^ bacteria/ml in Page’s saline solution (PAGE: 120 mg/l NaCl, 4 mg/l MgSO_4_ · 7H_2_O, 4 mg/l CaCl_2_ · 2H_2_O, 142 mg/l Na_2_HPO_4_ and 136 mg/l KH_2_PO_4_).

*Acanthamoeba polyphaga* (strain ATCC 50362) was grown overnight in peptone-yeast extract-glucose (PYG) medium [17]. The trophozoites were then washed three times and re-suspended in PAGE. Finally, the amoebae were counted and their concentration was adjusted to approximately 9 × 10^5^ cells/ml.

### Sterile compost and air samples

The compost was collected in an open-air composting facility in southern Switzerland. Compost samples were sterilized for 15 min at 121°C before spiking to eliminate *Legionella* cells potentially present in the compost [4].

Air samples are usually collected in the field with a portable cyclonic air sampler (Coriolis μ, Bertin technologies, Montigny, France) with a flow rate of 250 l/min during 4 minutes and the aspirate is diluted in 10 ml PAGE. Hence, for our experiments we used 10 ml sterilized PAGE samples spiked with known amounts of *Legionella* cells.

### Spiking of the compost and air samples

To assess the detection limits and the recovery efficiency of culture and co-culture, 9 aliquots of 5 g sterile compost or of 9 ml of sterile PAGE were spiked with 1 ml of serial dilutions of Lp1 suspension to obtain a dilution range of 1 to 1 × 10^8^ cells per 5 g of compost or per 10 ml PAGE. Ten millilitres of sterile PAGE or 5 g sterile compost re-suspended in 10 ml sterile PAGE were used as negative controls. After spiking, compost and PAGE were thoroughly mixed to distribute bacteria homogeneously in the samples and 9 ml of sterile PAGE were added to the compost. The compost suspensions were mixed during 30 min at room temperature.

### Recovery of *Legionella* from spiked samples by conventional culture

Ten microlitres of the compost supernatants and of the PAGE samples were diluted 1:100 with 0.2 M HCl-KCl acid buffer (pH 2.2), vortexed three times during 10 min and incubated at room temperature as previously described [18]. Then, 50 μl of each dilution and negative control of the spiked compost supernatant and PAGE samples were plated directly onto GVPC agar (bioMérieux) in duplicate and incubated at 36°C for 5 days. The amount of sample inoculated on the plate was 1/20,000 of the original compost portion.

### Recovery of *Legionella* from spiked samples by co-culture

Co-culture was performed using a PAGE suspension of axenic *A*. *polyphaga*. A suspension of 900 μl of amoebae (approximately 9 × 10^5^ amoebae/ml) was added to each well of a 24-well microplate (TPP, Techno Plastic Products AG, Trasadingen, Switzerland) and incubated for 1 h at 36°C to obtain an amoebal monolayer. One-hundred microlitres of each spiked compost supernatant were then added to each well. One well of each plate contained only a PAGE suspension of axenic *A. polyphaga* as negative control. After inoculation, the microplates were centrifuged at 1,000 g for 30 min and incubated during 7 days at 36°C in a moist chamber [12]. After 7 days the wells were scraped with a 1,000 ml pipette tip to detach the amoebal monolayer from the well bottom. Then, 20 μl samples were diluted 1:10 with 0.2 M HCl–KCl acid buffer (pH 2.2) and vortexed three times during 10 min at room temperature. After acid shock, 100 μl amount of each acid-treated sample was then plated on solid GVPC agar and incubated at 36°C for 5 days.

### Recovery of *Legionella* from untreated, natural samples

Culture and co-culture were performed in parallel on 88 compost and 23 air samples collected in composting facilities located in southern Switzerland. Air samples of 1 m^3^ were collected in 10 ml PAGE as previously described and compost samples were collected and stored in plastic bags at 4°C for 24 h. Compost supernatants were also plated directly onto both GVPC and MWY agar (bioMérieux). All *Legionella*-like colonies were identified by MALDI-TOF MS [1] and by slide agglutination tests (Legionella Slidex, bioMérieux, Switzerland). Serotyping of *Legionella pneumophila* isolates was performed by indirect immunofluorescence assay, using the monoclonal antibodies from the Dresden panel [19].

### Data analysis

Mean and standard deviations of the colony forming units (CFU) values obtained were determined in two parallel experiments for both compost and air samples. All measurements were carried out in duplicate. Calculations and graphical displays were prepared using Microsoft Excel 2003.

The limit of detection for direct culturing and co-culture of the spiked compost and air samples was defined as the fifth percentile of all analyzed positive and negative samples.

The final *Legionella* counts of both methods were multiplied by the corresponding dilution factor of each method to normalized the data. 100% efficiency of recovery was calculated as if all inoculated *Legionella* could be recovered.

## Results and discussion

This study demonstrates that the detection limit of co-culture is lower than that of conventional culture and allows detecting Lp1 in compost and air samples when the concentration is as low as 10^5^ cells in 1 m^3^ air and 10^6^ in 1 g compost samples.

**Figure 1:**
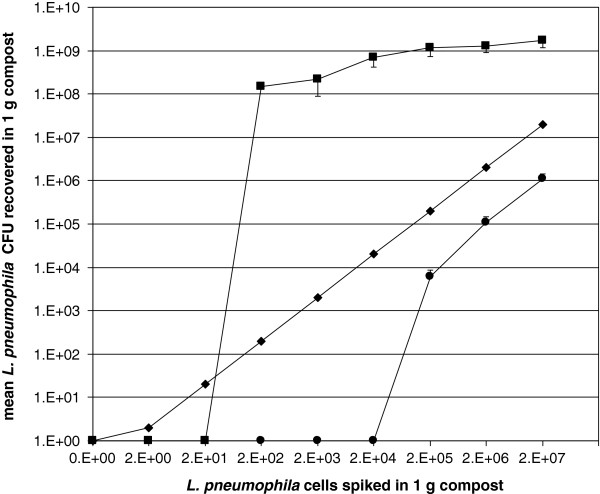
**Recovery of spiked *L. pneumophila *in sterilized compost sample.** (●) culture, (■) co-culture and (♦) theoretical recovery by 100% efficacy (means; bars: standard deviation).

**Figure 2:**
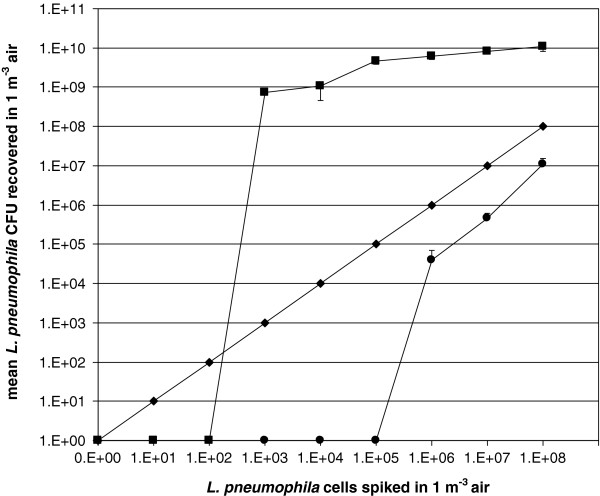
**Recovery of spiked *L. pneumophila *in sterilized air sample.** (●) culture, (■) co-culture and (♦) theoretical recovery by 100% efficacy (means; bars: standard deviation).

The recovery of Lp1 from the compost by co-culture was significantly higher than with culture alone: the co-culture method showed a 3 logs higher sensitivity, with a detection limit of 10^2^ in 1 g (culture: 10^5^ in 1 g compost) (Figure 1), similarly the recovery of Lp1 from the air (Figure 2) by co-culture was 3 log units higher, with a detection limit of 10^3^ Lp1 cells in 1 m^3^ air (culture: 10^6^ cells in 1 m^3^ air).

Recovery from air and compost samples by conventional culture were approximately one log unit lower, compared to the theoretical recovery by 100% efficiency. By contrast, the recovery by co-culture from both compost and air were at least 2 logs higher compared to the theoretical recovery by 100% efficiency (Figure 1 and Figure 2).

An important limitation of this, as well as of previous, similar studies, is the lack of quantification of the amplification power by amoebae. In fact, only *Legionella* cells that grow on GVPC agar after interaction with *A. polyphaga* can be counted. The amount of *Legionella* cells that are present as free cells in the supernatant and the cells that are not phagocyted by the amoeba cannot be assessed. Entry/uptake of *Legionella* by the amoebae, the ability of *Legionella* to replicate within and to escape from the amoebal cytoplasm cannot be reliably quantified using standard methods [20]. We further observed that co-culture needs longer incubation periods than culture. We do not tested the recovery of *Legionella* from spiked samples without acid treatment, we are aware that this causes a dilution of samples, but for non-sterile compost samples the recovery of *Legionella* without acid treatment is not possible due to overgrowth of contaminant flora. Nevertheless, our study shows that co-culture, on the average, allows detecting smaller amounts of *Legionella* cells in a given substrate.

The analysis of non-sterile compost samples with a higher load of *Legionella* contamination showed no relevant difference in isolation rates between culture and co-culture; by contrast, recovery of *Legionella* from air samples, in which a lower contamination load can be expected, was possible only by co-culture (Table 1). In the compost samples with negative co-culture the load of *Legionella* is high. In general, other non-*pneumophila* species and contaminant flora present in the non-sterile compost samples could compete with *Legionella* for amoebal uptake (Additional file 1). The hypotheses that could explain the negative co-culture results are: not all *Legionella* cells could replicate within amoebae and *Legionella* could “be eaten”; it is also possible that the cells were not uptake by the amoebae (no contact or interaction). It should be noted that the population of *Legionella* represent only the 0.01% of all the compost bacterial flora [21].

**Table 1:** **Table 1 Percentage and no. of samples from wich ***Legionella ***spp. were recovered by culture and co-culture**

	**Compost (n = 88)**		**Air (n = 23)**	
	**Culture**	**Co-culture**	**Culture**	**Co-culture**
Lp2-15	60.2% (53)	55.7% (49)	-	39.1% (9)
Lp1	25% (22)	11.4% (10)	-	8.7% (2)
Lp	6.8% (6)	3.4% (3)	-	-
*L. bozemanii*	39.8% (35)	6.8% (6)	-	4.3% (1)
*L. londiniensis*	26.1% (23)	-	-	-
*L. micdadei*	12.5% (11)	1.1% (1)	-	-
*L. oakridgensis*	11.4% (10)	-	-	-
*L. feeleii*	3.4% (3)	2.3% (2)	-	-
*L. jamestowniensis*	2.3% (2)	-	-	-
*L. birminghamensis*	1.1% (1)	-	-	-
*L. cincinnatiensis*	1.1% (1)	-	-	-
*L. sainthelensis*	1.1% (1)	-	-	-
*L. longbeachae*	-	1.1% (1)	-	-

Lp1: *L. pneumophila* serogroup 1; Lp2-15: *L. pneumophila* serogroups 2–15. Lspp: undetermined *Legionella* species.

Culture, however, yields apparently a better picture of the biodiversity of *Legionella* spp. in compost (Table 1); in fact, more species were recovered from each sample, whereas only one or two species per sample were enriched by co-culture (Additional file 1). Up to now, in Switzerland and in Europe mainly *L. pneumophila* was isolated from compost [4,22], in contrast to Australia and Japan where *L. longbeachae* was frequently isolated from compost by the conventional culture method [3,23].

Co-culture allowed enriching Lp1 by up to 6 log units from the starting bacterial cells number; the method is thus potentially useful in environmental monitoring, in particular when low *Legionella* loads are expected (e.g. bioaerosol, rain and water). The presumptive concentration of *Legionella* bacteria in the bioaerosols of composting facility is between 0 to 10^3^*Legionella* per m^3^.

The detection of *Legionella* in environmental samples such as soil and compost is hampered by the presence of other microorganisms (mould and bacteria) that grow on selective media and may interfere with the *Legionella* growth, leading to an underestimation of the effective number of *Legionella* present in the sample [4]. PCR allows quantification, but the amplification of DNA of dead cells present in a sample makes the interpretation of results difficult; PCR is not an alternative for a reliable quantification of *Legionella* in environmental samples because humic acids present in the samples may inhibit the reaction [24,25]. PCR has also been used to detect *Legionella* spp. in clinical samples, but sensitivity varies greatly (30-90%) depending on the type of specimen studied. In addition, the design of generic *Legionella* spp. primers is difficult [26].

Previous studies reported that the use of co-culture has allowed the isolation of *L. pneumophila* when plating on BCYE agar plates did not yield any colonies; it has also allowed isolation of several fastidious *Legionella* species from clinical stool [27] and sputum samples [12], as well as from environmental samples such as floating biofilms [28]. Co-culture allows the recovery of VBNC cells [14,29] or of some *Legionella* species not growing onto BCYE agar [12], such as *Legionella*-like amoebal pathogens (LLAP) [30] or *L. pneumophila* in pulmonary specimens [31]. According to Descours et al. (2012) the amoebic co-culture was effective to isolate *Legionella* spp. from respiratory samples contaminated with other microorganisms even if the type of sample impacted on the performance of culture and co-culture [31].

## Conclusions

The use of co-culture is thus potentially useful to detect *Legionella* spp. in clinical samples with a low degree of contamination by *Legionella* spp., but the long incubation period needed is a strong negative aspect of the method.

Further studies are needed to test different amoebal strains susceptibilities to various *Legionella* species. The detection of *Legionella* in environmental samples is still commonly carried out by conventional culture, but co-culture should be considered whenever there is a need to detect *Legionella* or VBNC expected to be present at concentrations below 10^5^ – 10^6^ cells, in particular when working with air samples.

## Competing interests

The authors declare that they have no competing interests.

## Authors’ contributions

LC, SC and VG participated in the conception and design of the study and participated in the analysis and interpretation of data. LC wrote the first draft of the manuscript which was extensively reviewed by SC and VG. All authors have read and approved the final manuscript.

## Supplementary Material

Additional file 1 **xls List of all *Legionella *spp. recovered from non-sterile compost (88) and air (23) samples analysed in parallel by culture and co-culture.** Lp1: *L. pneumophila* serogroup 1; Lp2-15: *L. pneumophila* serogroups 2–15; Lspp: undetermined *Legionella* species; *non-*Legionella* species recovered by co-culture. Click here for file
